# Role of the police in linking individuals experiencing mental health crises with mental health services

**DOI:** 10.1186/1471-244X-12-171

**Published:** 2012-10-17

**Authors:** Rob HS van den Brink, Jan Broer, Alfons J Tholen, Wim H Winthorst, Ellen Visser, Durk Wiersma

**Affiliations:** 1 Department of Psychiatry, University Medical Center Groningen, University of Groningen, Groningen, The Netherlands; 2 Municipal Health Service Groningen, Groningen, The Netherlands; 3 Lentis Mental Health Organisation (affiliation at time of study), Groningen, The Netherlands; 4 Psychiatric Case Register North Netherlands, University Medical Center Groningen, Groningen, The Netherlands

**Keywords:** Mental Health Services, Utilisation, Crisis intervention, Police

## Abstract

**Background:**

The police are considered frontline professionals in managing individuals experiencing mental health crises. This study examines the extent to which these individuals are disconnected from mental health services, and whether the police response has an influence on re-establishing contact.

**Methods:**

Police records were searched for calls regarding individuals with acute mental health needs and police handling of these calls. Mental healthcare contact data were retrieved from a Psychiatric Case Register.

**Results:**

The police were called upon for mental health crisis situations 492 times within the study year, involving 336 individuals (i.e. 1.7 per 1000 inhabitants per year). Half of these individuals (N=162) were disengaged from mental health services, lacking regular care contact in the year prior to the crisis (apart from contact for crisis intervention). In the month following the crisis, 21% of those who were previously disengaged from services had regular care contact, and this was more frequent (49%) if the police had contacted the mental health services during the crisis. The influence of police referral to the services was still present the following year. However, for the majority (58%) of disengaged individuals police did not contact the mental health services at the time of crisis.

**Conclusions:**

The police deal with a substantial number of individuals experiencing a mental health crisis, half of whom are out of contact with mental health services, and police play an important role in linking these individuals to services. Training police officers to recognise and handle mental health crises, and implementing practical models of cooperation between the police and mental health services in dealing with such crises may further improve police referral of individuals disengaged from mental health services.

## Background

The police are often called upon to attend to individuals experiencing mental health crises [1,2]. For example, the police may be asked to intervene if individuals are endangering their own lives, fouling their homes, wandering the streets in a state of confusion, or disturbing their neighbours. These situations often involve individuals who experience severe mental health problems, for which they do not seek help, but who are judged by others to be in need of help, and hence are considered a public concern. We therefore refer to these situations as public mental health crises. In many countries, the police are legally obliged to offer help to citizens who are in need of assistance but cannot care for themselves [2].

With the deinstitutionalisation of mental health services, an increasing number of individuals with severe mental health problems live in the community. Many of these individuals are not, or no longer, in contact with mental health services for regular care, and may only receive acute care, if any, in crisis situations [3]. Such individuals may come to the attention of the police if they experience a mental health crisis, together with individuals who receive regular mental health care, but for whom a crisis could not be prevented. According to Lamb et al. [2], the police therefore have thus become ‘frontline professionals’ in managing individuals who experience a mental health crisis.

Little is known about the nature of the mental health problems faced by people who are managed by the police during crises, or the way in which the police deal with these crises. Several studies investigated patients referred by the police to psychiatric emergency services e.g. [4,5], but it remains unclear what proportion and selection this constitutes of all individuals seen by the police for mental health crises [5], or what other strategies the police use to deal with these crises. Research into the police response to mental health crises has typically focused on diversion from the criminal justice system [6-8] rather than the effectiveness of the police response in establishing contact between patients and mental health services.

A major obstacle in studying public mental health crises is that police information and mental health care information about individuals are usually kept strictly separated [9]. We received permission to link police and mental health care information to evaluate a 24/7 drop-off centre at the police station for people experiencing mental health crises [10], providing a unique opportunity to examine these crisis situations.

The present study sought to determine the types of mental health problems experienced by individuals seen by the police for mental health crises, the extent to which these individuals were in contact with mental health services at the time of crisis, the ways in which the police handle mental health crises, whether this has an effect on contact with mental health care after the crisis, and whether this effect is long-lasting.

## Methods

### Study design

Records of an urban police district in the Netherlands were searched for calls to the police regarding mental health crises in one year. The police response to the crisis was assessed from the written account of the incident by the police officer who responded to the call. We searched for the individuals involved in a regional Psychiatric Case Register that records all contact between clients and local mental health services. Diagnostic information and the number of care contacts were retrieved. For individuals with multiple mental health crises during the study period, only the first crisis was included. We sought to establish: (1) the number of individuals without regular care contact (i.e. contact other than for crisis intervention) in the year before the crisis, (2) the relationship between the police response to the crisis and any increase in number of regular care contacts from the month before to the month after the crisis, and (3) for those who did not have contact with care in the year before the crisis, the relationship between the police response and the likelihood of care contact in the month following the crisis, and in the subsequent year.

The study was approved by the Ministry of Justice and the Psychiatric Case Register has been reported to the Data Protection Authority. Information that could identify an individual was excluded from the study database.

### Selection of calls for mental health crises

The database of the police district of Groningen (198,000 inhabitants) was searched for reports of encounters with individuals with acute mental health needs, between April 1, 2003 and April 1, 2004. All incoming calls to the police are registered and, if responded to, the responding officer is required to enter a report into the database. The report consists of a non-standardised description of the encounter by the police officer, which is categorised by the officer into a limited number of ‘incident codes’. Included in this study were all reports with the codes ‘Nuisance by a presumably disturbed person’ and ‘Suicide attempt’. In addition, the text of reports under the codes ‘Assistance of citizen’, ‘Domestic or neighbours’ quarrel’, and ‘Violence’ were searched for key words indicating a mental health need, such as ‘confused’, ‘disturbed’, ‘mental’, ‘off the handle’, etc. These latter reports were only included if they described a person with clear mental health problems, in acute need of care. We excluded reports of persons who were arrested for an offence, were classified as ‘Alcohol abuse’ or ‘Drug abuse’, and reports describing calls that were not responded to or in which the police did not find the person concerned. People who were taken into custody were excluded because we wanted to focus on situations in which it was clear that the police had the option to link the person to mental health services at the time of crisis. Cases of alcohol and drug abuse were excluded because intoxicated persons are typically taken to the police station to sober up, and are sent home after several hours with a fine but no care assistance [10].

### Police response to the crisis

The police response to the crisis was assessed from the non-standardised report of the incident by the responding officer. The response was categorised as: (1) dealt with by the police, without contacting care; (2) mental health services (including addiction services) contacted by the police; (3) other care services contacted by the police; or (4) person taken to the crisis drop-off centre at the police station for a mental health evaluation, in collaboration with the mental health services.

### Clients’ contact with care services

We examined the regional Psychiatric Case Register for information about the individuals seen by the police for mental health crises [11]. This register records all cases of mental health care contact in the wider region of the police district [12]. Only cases of ‘regular care contact’ were studied (i.e. excluding contact relating solely to a crisis situation), because we wanted to distinguish clients in regular care from those who only come in contact with services at times of crisis. The number of regular mental health care contacts in the year before the crisis was classified into: (1) frequent care contacts, i.e. three or more inpatient days or ten or more outpatient contacts; (2) some care contacts, i.e. up to two inpatient days and up to nine outpatient contacts; and (3) no care contact. In addition, we established the numbers of regular care contacts for three periods: the month before the crisis, the month after the crisis, and the subsequent year (i.e. 1 to 13 month after the crisis). Here no distinction was made between inpatient days and outpatient contacts, counting both as a single contact.

### Diagnostic information

Information about the nature of the mental health problems of individuals experiencing public mental health crises was gathered from the following sources, in order of preference: (1) the mental health evaluation at the time of crisis at the drop-off centre at the police station, with access to patient files of the mental health services; (2) diagnostic information in the Psychiatric Case Register, obtained at contacts with mental health services; and (3) the police report of the crisis. Not all sources provided a clinical diagnosis. Hence, the classification of diagnostic information constitutes a classification of mental health problems, not of mental disorders.

### Characteristics of mental health calls

Characteristics of the mental health calls were obtained from the police report of the call. In addition, the nature of the crisis situation was judged from the report and categorised as danger to self, danger to others, self-neglect, or nuisance.

### Analysis

The relationship between the type of police response and care contact after the crisis was studied in two ways. First, for all individuals the influence of the police response on the change in number of contacts from the month before to the month after the crisis was examined using an analysis of variance with post-hoc t-tests between individual responses. Because prior contact with mental health services may facilitate an increase in contact after the crisis, we also tested whether the influence of the police response depends on the level of contact the person had with the services in the year before the crisis. Second, for individuals who did not have regular care contact with services in the year before the crisis, we tested whether the police response was related to the likelihood of having regular care contact in the month after the crisis, i.e. how the police response was related to ‘linking’ these individuals to relevant services. Finally we tested whether this linking to services resulted in lasting care relationships that persisted into the following year. These effects were tested using overall Chi-square tests and post-hoc tests for differences between individual police responses.

## Results

### Mental health-related calls to the police

During the one-year study period, 492 calls related to mental health crises were identified in the police database. With a population of 198,000 inhabitants in the police district, this constitutes a rate of 2.5 calls per 1000 inhabitants per year. Table 1 presents characteristics of the mental health calls received by the police. Importantly, in one-fifth of the calls it was the individual experiencing the crisis who contacted the police. In addition, in approximately half of the cases, the crisis was judged to constitute a problem to others (46%: nuisance or danger to others), while only a quarter of the cases were deemed to constitute a problem for the individual experiencing the crisis (23%: self-neglect or danger to self).

**Table 1:** Characteristics of mental health calls to the police (N=492)

**Characteristics**	**N**	**%**
**Caller**		
Neighbour or bystander	167	34
Agency (nuisance complaints desk; pension; etc.)	95	19
Person self	93	19
Family or friend	75	15
Victim or police surveillance	30	6
Unclear or missing	32	7
Time of call		
Working day; outside office hours	191	39
Working day; during office hours	176	36
Weekend or holiday	125	25
Location where person was found		
Public space	220	45
At home	184	37
Unclear or missing	88	18
Police incident code		
Nuisance by disturbed person	250	51
Assistance of citizen	161	33
Domestic or neighbours’ quarrel	34	7
Suicide attempt	18	4
Violence	13	3
Missing	16	3
Nature of mental health crisis		
Nuisance to others	178	36
Danger to self	108	22
Danger to others	48	10
Self-neglect	5	1
Other or unclear	153	31

### Individuals experiencing public mental health crises

The 492 mental health calls concerned 336 individuals (1.7 per 1000 inhabitants per year). Their mean age was 40.1 years (SD=16.3), ranging from 13 to 93 years. Sixty percent of these individuals were male, and 18% were homeless. For 74 individuals there were multiple calls (range 2–11) within the study year.

Fifteen individuals came from outside the region covered by the Psychiatric Case Register, meaning there was no information on their contact with mental health services. These individuals were excluded from further analyses. Of the remaining 321 individuals, 162 (50%) did not have regular mental health care contact in the year before the crisis, 52 (16%) had some care contacts (up to two inpatient days and up to nine outpatient contacts), and 107 (33%) had frequent care contacts (three or more inpatient days or ten or more outpatient contacts).

Table 2 shows the nature of the mental health problems experienced by individuals with and without care contact in the preceding year. Due to the lack of contact with mental health services, no formal diagnosis was available for 36% of individuals without regular care contact in the year before the crisis. Nevertheless, the diagnoses that were available (from crisis interventions, or care contact prior to a year before the crisis, or contact after the crisis) revealed that a substantial portion of individuals seen by the police for mental health crises experience serious mental health problems that warrant referral to mental health services. For example, at least 26% of individuals without care contact had a psychotic or bipolar disorder, which are generally regarded as serious mental illnesses, and another 30% (controlling for comorbidity with psychotic or bipolar disorders) had a potentially serious cognitive, substance use or depressive disorder. For individuals with care contact, these figures were 45% and 36%, respectively.

**Table 2:** Mental health problems of individuals with public mental health crises

Mental health problems^*^	**With care contact**		**Without care contact**	
	**(N=159)**		**(N=162)**	
	N	%	N	%
Psychotic disorder	59	37	33	20
Bipolar disorder	13	8	10	6
Other mood disorder	15	9	5	3
Dementia or other cognitive disorder	3	2	11	7
Substance use disorder	75	47	52	32
Personality disorder	49	31	14	9
Other mental disorder	44	28	22	14
Unclear mental health problem^#^	9	6	59	36

*Classification into multiple categories for comorbid disorders.

#Mental health problem described, but no diagnosis provided.

### Police response to mental health crises

The frequencies of different types of police response to a mental health crisis are presented in the left panel of Table 3. Only the responses to the first crisis per individual in the study period are shown, but an almost identical pattern of results was observed for all crises. In approximately half of the cases the police dealt with the crisis themselves, without contacting any care services (e.g. by calming the individual or contacting a relative to look after them). In 27% of crises the police contacted mental health services (e.g. the client’s case manager or a psychiatric emergency service), and in 10% the police contacted another care service (e.g. the person’s general practitioner or social services). Finally, 14% of individuals were taken to the crisis drop-off centre at the police station, for a mental health evaluation by a public health physician of the municipality or a professional of the mental health services. This option was often chosen outside office hours and for clients who did not live in the area or were homeless [10]. The 24/7 crisis drop-off centre therefore appears to be used by police officers as an additional entry point to mental health services, at times when access to these services is difficult [10]. 

**Table 3:** Police response to mental health crises and relationship with care contacts^*^

**Police response to crisis**	**All persons (N=321)**				**Persons without care contact in year before crisis (N=162)**					
	**Frequency of response**		**Increase in number of contacts per month**^**#**^		**Frequency of response**		**Persons with contact in month after crisis**		**Persons with contact in subsequent year**	
	**N**	**%**	**Mean**	**SD**	**N**	**%**	**N**	**%**	**N**	**%**
Dealt with by police	156	49	1.3^¶^	5.9	94	58	5	5^§^	19	20
Mental healthcare contacted	88	27	7.4^¶^	12.6	29	18	12	41^§^	17	59
Evaluation at police station	44	14	7.0^¶^	10.8	20	12	12	60^§$^	12	60
Other care contacted	33	10	4.0	8.9	19	12	5	26^§$^	7	37

*Only first crisis in study period for each individual.

#Increase in care contacts from month before to month after crisis.

¶Row 1 different from rows 2 and 3 (p<.01).

§Row 1 different from all other rows (p<.01).

$Row 3 different from row 4 (p=.03).

### Relationship between police response and number of care contacts

The mean number of regular care contacts increased from 3.4 (SD=8.4) in the month before the crisis to 7.4 (SD=11.6) in the month after the crisis (paired t=7.53, df=320, p<.01), when individuals were seen for the first time in the study period. No interaction effect was found between the type of police response and the number of care contacts in the year before the crisis on the increase in number of contacts after the crisis (F=0.61, df=6;309, p=.72). In addition, there was no main effect of the number of care contacts on the increase in contacts (F=0.82, df=2;309, p=.44). However, the increase in the number of care contacts from the month before to the month after the crisis was related to the type of police response to the crisis (F=7.23, df=3;309, p=<.01), as illustrated in the left panel of Table 3. A greater increase was observed when police had contacted mental health services during the crisis or had taken the person to the crisis drop-off centre, compared with situations where crises were dealt with by the police themselves (t=5.13, df=242, p<.01 and t=4.66, df=198, p<.01, respectively).

### Linking individuals to services

Of special interest is the effect of the police response for individuals with no regular care contact with mental health services prior to their mental health crisis. We tested whether the way in which the police handled the crisis affected the likelihood of regular care contact (the individual’s linkage with mental health services) after the crisis.

Of the 162 individuals without contact with mental health services in the year before the crisis, 34 (21%) had regular care contact (other than for crisis intervention) with these services in the month after the crisis. In addition, the likelihood of care contact was related to the police response to the crisis (χ^2^=39.87, df=3, p<.01), as shown in the middle panel of Table 3. This likelihood was greater for individuals who were taken to the crisis drop-off centre or for whom the police directly contacted the mental health or another care service during the crisis, compared with those for whom the police did not contact a service to deal with the crisis (χ^2^=38.86, 24.20 and 8.64, respectively, all df=1, p<.01). Taking the individual to the crisis drop-off centre was also more effective than contacting another care service for linking the person to mental health services (χ^2^=4.50, df=1, p=.03). Importantly, the least effective response for linking individuals to mental health services was chosen most frequently by the police. In 58% of mental health crises of persons who were out of contact with services, the police dealt with the crisis themselves and did not contact a service provider.

Of the 34 individuals who were newly linked to the services, 25 were admitted (admission was compulsory for 12 individuals) to a psychiatric hospital in the month after the crisis, for 13.2 days on average (excluding the crisis day; SD=12.3; range 1–30), and the remaining nine had 1.4 outpatient contacts on average (SD=0.7; range 1–3).

Finally, we examined whether individuals who were newly linked to mental health services in the month after the crisis developed a lasting care relationship with the services that continued into the subsequent year. The right panel of Table 3 shows the number of individuals who had regular care contact with mental health services in the period from 1 to 13 months after the crisis. For all persons who were disengaged from the services before the crisis, this number increased from 34 (21%) in the first month after the crisis to 55 (34%) in the subsequent year. Again, this number was related to the police response to the crisis (χ^2^=21.91, df=3, p<.01). When police contacted mental health services at the time of crisis, either directly or by taking the person to the crisis drop-off centre, individuals were more likely to develop a lasting care relationship with services, compared with individuals whose crisis was dealt with by the police themselves without contacting service providers (χ^2^=15.79 and 13.19, respectively, both df=1, p<.01). Furthermore, this relationship more often consisted of frequent care contacts (i.e. three or more inpatient days or ten or more outpatient contacts) for individuals referred to mental health services directly or indirectly, compared with individuals dealt with by the police only (39% versus 12%; χ^2^=14.24, df=1, p<.01, data not shown). For five of the 34 individuals who were newly linked to mental health services in the first month after the crisis, the care relationship did not persist into the subsequent year, and this was not related to the police response to the crisis (χ^2^=0.65, df=3, p=.89).

## Discussion

The present study revealed that the police play an important role in referring people to mental healthcare. They come into contact with a large number of individuals experiencing a mental health crisis (1.7 per 1000 inhabitants per year) Of these individuals, half are not in contact with mental health services at the time of crisis. Police are responsible for bringing a substantial portion of these individuals (21%) into contact with mental health services. Furthermore, the likelihood of mental health care contact after the crisis and of sustained utilisation of mental health care are related to whether or not the police contact mental health services at the time of crisis.

The current results do not necessarily imply a causal relationship between police response to the crisis and subsequent care contact. It is possible that contact would have occurred anyway, irrespective of the police response. For example, the severity of certain mental health problems might make (compulsory) contact with mental health services more or less inevitable. However, the police can facilitate this contact by taking the necessary first step in contacting mental health services at the time of crisis, preventing further delay in the provision of care.

Several studies have examined the police response to mentally ill individuals [13,14]. These studies differ from the present report in the way police calls were selected for study. Nevertheless, the results are in accord with the current findings. Police contact mental health services in approximately half of all encounters with individuals experiencing mental health crises, and deal with the other half themselves.

Both Watson et al. [13] and Teller et al. [14] reported that police officers who were trained in recognising and dealing with persons with mental health problems, in accordance with the prevailing Crisis Intervention Team (CIT) model in the United States, directed a greater proportion of mentally ill persons to mental health services compared with their non-trained colleagues. However, this is not necessarily an effect of training, since officers volunteer to participate in CIT and are screened to determine their suitability [15,16]. CIT-officers may therefore already be more open to the notion of referring individuals to mental health services, and were found to be older and more experienced than their non-volunteering peers [13]. In addition, mental health calls may be allocated selectively to CIT-trained versus non-CIT trained officers. CIT therefore represents a potentially important model for the police handling of mental disturbance calls, alongside other models such as mobile crisis teams staffed by both police officers and mental health professionals [17,18], but the effect of CIT training on the referral of mentally ill individuals to mental health services remains uncertain.

### Study strengths and limitations

Obtaining permission to link police and mental healthcare information provided a unique opportunity to study the linkage with mental health services of individuals seen by the police for mental health crises. Furthermore, the current study was unique in examining the effectiveness of the police response to the crisis in establishing (sustained) contact of mentally ill individuals with mental health services. However, several limitations of the study should be considered. First, mental health crises were not specifically indicated in the police database we studied, but were identified by a search procedure based on the labelling of calls by police officers, key words used in reports, and an evaluation against our definition of mental health crises. This selection method may have introduced some ambiguity in the identification process. Second, the availability of professional information on the nature of the mental health problems differed markedly between subjects, depending on their contact with mental health services. Third, the Psychiatric Case Register may not have covered all mental health care contacts of the individuals experiencing mental health crises. For example, the register does not include contact with mental health services outside the region, contact with private mental health practices, or contact with prison mental health services. Finally, although the results revealed that police referral to the services at the time of crisis was associated with increased and sustained utilisation of mental health care, the limited period of police data prevented us from determining whether this improved care also reduced the chance of new crisis contacts with the police.

## Conclusions

Despite the limitations described above, the present study supports the notion that the police are important ‘frontline professionals’ for individuals who experience a mental health crisis [2], and that the police response to these crises is crucial in linking individuals to mental health services. The competence of police officers in recognising and handling mental health crises is therefore rightly stressed in the literature [14,19], as is the importance of practical models of cooperation between the police and mental health services in dealing with these crises [1,10,20,21]. However, the present results also indicate that there may be room for improvement, as more than half of the individuals who were disengaged from mental health services were not connected with services at the time of crisis. This finding reinforces the notion that the contribution of the police to the identification and referral of individuals with mental health problems deserves serious study, both of the current contribution and of the potential contribution in the future.

## Competing interests

The authors have no competing interests.

## Authors’ contributions

RHSVDB, JB, AJT, WHW and DW participated in the design of the study. Data collection was performed by RHSVDB, JB, AJT, WHW and EV. RHSVDB and JB analysed the data. All authors were involved in interpretation of the data and revision of the manuscript. The final version of the manuscript was approved by all authors.

## Pre-publication history

The pre-publication history for this paper can be accessed here:

http://www.biomedcentral.com/1471-244X/12/171/prepub
